# Ethical considerations in deploying triple artemisinin-based combination therapies for malaria: An analysis of stakeholders’ perspectives in Burkina Faso and Nigeria

**DOI:** 10.1371/journal.pone.0273249

**Published:** 2022-09-09

**Authors:** Paulina Tindana, Rosemonde Guissou, Oladimeji Akeem Bolarinwa, Fatoumata Tou, Freek de Haan, Mehul Dhorda, Arjen M. Dondorp, Chanaki Amaratunga, Olugbenga Ayodeji Mokuolu, Jean Bosco Ouedraogo, Phaik Yeong Cheah

**Affiliations:** 1 Department of Health Policy, Planning and Management, School of Public Health, College of Health Sciences, University of Ghana, Accra, Ghana; 2 Institut de Recherche en Sciences de la Sante, Bobo-Dioulasso, Burkina Faso; 3 Department of Epidemiology and Community Health, University of Ilorin, Ilorin, Nigeria; 4 Institut des Sciences et Techniques, Bobo-Dioulasso, Burkina Faso; 5 Copernicus Institute of Sustainable Development, Utrecht University, Utrecht, the Netherlands; 6 Mahidol Oxford Tropical Medicine Research Unit, Mahidol University, Bangkok, Thailand; 7 Nuffield Department of Medicine, Centre for Tropical Medicine and Global Health, University of Oxford, Oxford, United Kingdom; 8 Department of Paediatrics, University of Ilorin, Ilorin, Nigeria; 9 Nuffield Department of Population Health, The Ethox Centre, University of Oxford, Oxford, United Kingdom; Menzies School of Health Research, AUSTRALIA

## Abstract

**Background:**

Artemisinin-based combination therapies (ACTs) are the recommended treatment for uncomplicated *Plasmodium falciparum* malaria in all malaria endemic countries. Artemisinin resistance, partner drug resistance, and subsequent ACT failure are widespread in Southeast Asia. The more recent independent emergence of artemisinin resistance in Africa is alarming. In response, triple artemisinin-based combination therapies (TACTs) are being developed to mitigate the risks associated with increasing drug resistance. Since ACTs are still effective in Africa, where malaria is mainly a paediatric disease, the potential deployment of TACTs raises important ethical questions. This paper presents an analysis of stakeholders’ perspectives regarding key ethical considerations to be considered in the deployment of TACTs in Africa provided they are found to be safe, well-tolerated and effective for the treatment of uncomplicated malaria.

**Methods:**

We conducted a qualitative study in Burkina Faso and Nigeria assessing stakeholders’ (policy makers, suppliers and end-users) perspectives on ethical issues regarding the potential future deployment of TACTs through 68 in-depth interviews and 11 focus group discussions.

**Findings:**

Some respondents suggested that there should be evidence of local artemisinin resistance before they consider deploying TACTs, while others suggested that TACTs should be deployed to protect the efficacy of current ACTs. Respondents suggested that additional side effects of TACTs compared to ACTs should be minimal and the cost of TACTs to end-users should not be higher than the cost of current ACTs. There was some disagreement among respondents regarding whether patients should have a choice of treatment options between ACTs and TACTs or only have TACTs available, while ACTs are still effective. The study also suggests that community, public and stakeholder engagement activities are essential to support the introduction and effective uptake of TACTs.

**Conclusion:**

Addressing ethical issues regarding TACTs and engaging early with stakeholders will be important for their potential deployment in Africa.

## Introduction

Drug resistance is a major threat to global malaria control and elimination. Africa remains the continent with the highest malaria morbidity and mortality rates; and accounted for approximately 94% of global malaria cases in 2020 [1]. In the early 2000s, following widespread resistance resulting in treatment failures to conventional monotherapies, the World Health Organisation (WHO) recommended artemisinin-based combination therapies (ACTs) as first-line treatment for uncomplicated falciparum malaria [2]. Despite initial delays, African countries changed their malaria treatment policies to ACTs from 2004 onwards [3–5], and these ACTs have largely remained effective for the treatment of uncomplicated *Plasmodium falciparum* malaria in African countries. In most African countries, current first-line therapies are artemether-lumefantrine (AL) and artesunate-amodiaquine (ASAQ) [2].

Although ACT treatment failures have not been confirmed in Africa yet, artemisinin partial resistance in Africa has been reported [6–11]. Uwimana et al identified *Pfkelch13 561H* mutations associated with artemisinin partial resistance in Rwanda, corresponding to delayed parasite clearance [7, 10]. Asua et al identified *Pfkelch13 675V* mutations associated with artemisinin partial resistance in Uganda [6] and very recently Balikagala et al showed that this mutation and the *Pfkelch13 469Y* mutation were also associated with delayed parasite clearance in Uganda [11]. There have also been recent reports of possible AL failure in Burkina Faso and Angola, which could be related to partial lumefantrine resistance [12–15]. This has prompted calls for urgent action to prevent or delay the emergence of artemisinin and partner drug resistance in African countries. One proposed strategy is the use of triple artemisinin-based combination therapies (TACTs) [16–21].

The Development of Triple Artemisinin-based Combination Therapies (DeTACT) project includes the conduct of a randomized, placebo-controlled clinical trial in 3 Asian and 8 African countries to compare the safety, tolerability and efficacy of two TACTs against their matching ACTs (ClinicalTrials.gov identifiers NCT03923725 and NCT03939104) [16]. TACTs combine existing ACTs with a third currently available antimalarial drug. The TACTs, artemether-lumefantrine plus amodiaquine (AL+AQ) and artesunate-mefloquine plus piperaquine (ASMQ+PPQ) are currently being evaluated in the DeTACT project. Preceding studies showed that AL+AQ and dihydroartemisinin-piperaquine+mefloquine (DHA-PPQ+MQ) were safe, well-tolerated and highly effective against multidrug-resistant malaria in Asia [22]. The DeTACT project aims to have fixed-dose combination TACTs ready for deployment in early 2023.

However, there are key ethical considerations regarding the potential deployment and effective uptake of TACTs in Africa, where malaria is mainly a paediatrics disease. Currently, conventional ACTs are recommended both in national treatment guidelines and in the normative WHO guidelines [2]. Given that ACTs are still largely effective in Africa, an important ethical question is; ‘*what are the ethical considerations for switching to TACTs as a first-line therapy for the treatment of malaria*?*’*. While widespread deployment of TACTs could potentially benefit communities and populations by preventing or delaying artemisinin resistance, there is no immediate *additional* benefit to individual patients in regions where ACTs are still fully effective. However, given the recent reports of artemisinin partial resistance in Africa, more African countries could encounter increasing artemisinin and ACT partner drug resistance before TACTs are introduced. The scenario that ACTs remain effective at the moment of deployment of TACTs implies that paediatric patients would be required to carry an additional burden (associated with taking an additional antimalarial drug in the TACT combination) for the public good and in the interest of future populations with falciparum malaria. The current study sought to identify potential ethical issues related to the deployment of TACTs in children with falciparum malaria in a context where TACTs have shown to be cost-effective and delay drug resistance, but can cause additional or increased side effects. The key ethical issues identified previously for the deployment of TACTs in Africa include patient (or parent) autonomy, balancing the potential additional harms and burdens of TACTs to patients versus public good, while securing the best interests of the child [23].

Approaching these issues through a public health ethics lens and drawing on ethics frameworks such as those proposed by Upshur [24], Childress et al [25] and Kass [26], raises the question whether or not it is ethically justifiable to override other moral considerations to individuals such as patient autonomy to promote the goals of public health. These public health ethics frameworks suggest that potential burdens and harms that could arise from public health programmes are identified and anticipated in advance and are minimized [24–26]. If the public health goal of TACTs is to mitigate the risk of drug resistance and protect the therapeutic efficacy of ACTs, thereby preventing malaria morbidity and mortality across Africa and the rest of the world, is it justifiable to ask patients to carry an additional burden associated with TACT treatments for the sake of public good? Beyond justifying the public health benefits of deploying TACTs in Africa, given previous experiences with the slow change of national malaria policies in the early 2000s, it is crucial that important ethical issues are addressed before the deployment of TACTs, and that these are informed by the perspectives of stakeholders such as policy makers, implementers, prescribers and end-users [27].

In this paper, we report findings of a qualitative study on the key ethical issues that should be anticipated to inform the effective *potential* deployment of TACTs in Africa. The analyses of the readiness of antimalarial markets in African countries for a transition to TACTs based on this qualitative study has been reported elsewhere [28]. Drawing on existing public health ethics frameworks and empirical qualitative data from in-depth interviews and focus group discussions in Nigeria and Burkina Faso, we discuss the *ethical considerations* for deploying TACTs in Africa and highlight the recommendations suggested by the stakeholders.

## Methods

### Study design

This study was an exploratory qualitative research study conducted as part of the Development of Triple Artemisinin-based Combination Therapies (DeTACT) project [16]. It involved the collection and analysis of stakeholders’ perspectives obtained through in-depth interviews (IDIs) and focus group discussions (FGDs) [27]. The study design was selected to elicit perspectives of diverse stakeholder groups within the healthcare service delivery. The use of open-ended questions for the interviews allowed participants to provide detailed information on the research topics based on their previous experiences.

### Study settings

The study was conducted in two countries in West Africa: Burkina Faso and Nigeria. Burkina Faso is a French-speaking country with a population of ~20 million people, while Nigeria is an English-speaking country and is the most populous country in Africa with a population of ~200 million (2019 population projections). According to the 2021 World Malaria Report, Nigeria and Burkina Faso accounted for 27% and 3.4% of the malaria cases globally in 2020 [1]. Both countries changed their malaria drug policy from sulfadoxine-pyrimethamine (Burkina Faso) and chloroquine (Nigeria) to ACTs in 2005 with AL and ASAQ as first-line treatments for malaria in both countries [29–31]. This context provided a unique opportunity to address the key research questions of this study through perspectives of those who are likely to be affected either as policy makers, suppliers or end-users should TACTs be deployed in a context of increasing antimalarial drug resistance.

### Sample selection

Three categories of respondents were targeted for this study in order to represent the various levels of the healthcare system: policy makers, suppliers and end-users. The respondents were then purposively selected to include these three levels of individuals (Table 1). Each country had a country-specific team of social scientists with experience in qualitative research and malaria research. They were fluent in the local languages and were responsible for identifying potential respondents, conducting interviews and performing preliminary analysis of the data.

**Table 1:** Stakeholder groups interviewed in Nigeria and Burkina Faso and type of interviews (IDI or FGD).

Country	Category of stakeholders	Number	Background / affiliation	IDI/FGD
**Nigeria**	**Policy makers**	1	Principal malaria researcher	IDI
		3	Regulatory officials	IDI
		6	National malaria policy officials	IDI
		1	Regional malaria policy official	IDI
	**Suppliers**	4	Public sector prescribers	IDI
		4	Private sector prescribers	IDI
		3	Village health workers	FGD
		2	Public sector wholesalers/retailers	IDI
		5	Private sector wholesalers/ retailers	IDI
	**End-users**	7	Community members	IDI
		4	Community members	FGD
**Burkina Faso**	**Policy makers**	2	Principal malaria researchers	IDI
		2	Regulatory officials	IDI
		3	National malaria policy officials	IDI
		3	Regional malaria policy officials	IDI
	**Suppliers**	5	Public sector prescribers	IDI
		9	Private sector prescribers	IDI
		3	Public sector wholesalers /retailers	IDI
		8	Private sector wholesalers/ retailers	IDI
	**End-users**	4	Community members	FGD

### Data collection

Data were collected in Nigeria from December 2019 to February 2020 and in Burkina Faso from March 2020 to June 2020. Prior to data collection, an initial team meeting was held in Ouagadougou, Burkina Faso in May 2019 to review and finalize the interview/discussion guides for the IDIs and FGDs [32]. The meeting also included discussion regarding the implementation of the project in both countries and how to purposefully select the appropriate stakeholders for IDIs and FGDs.

In Nigeria, data were collected in Abuja, the federal capital with national policy-level stakeholders and regulatory authorities while IDIs and FGDs with other stakeholders including clinicians and community members were conducted in Ilorin, Kwara state in the North-Central region of Nigeria. A total of 32 IDIs and 7 FGDs were conducted in Nigeria. In Burkina Faso, 35 IDIs and 4 FGDs were conducted in Ouagadougou (the capital), Bobo-Dioulasso and the village of Santidougou. A total of 68 IDIs and 11 FGDs were conducted in both countries (Table 1).

Each participant was taken through the informed consent process before conducting the IDIs or FGDs. For IDIs and FGDs conducted at the community level, permission was requested from the relevant community gate keepers before individuals were approached. Participants were assured of confidentiality of the data provided and informed that no personal identifiers would be included in the study reports or manuscripts. All participants have been given a unique identifier as reflected in the quotes used to support the key findings of the study.

### Data analysis

All IDIs and FGDs were audio-recorded and transcribed verbatim. In Nigeria most of the interviews were conducted in English. Those conducted among rural end-users at the community level in Kwara, Nigeria, were translated from the local language into English during the transcription process. All IDIs and FGDs in Burkina Faso were conducted in French and translated to English. The transcripts were uploaded to NVivo 12 qualitative analysis software to aid the coding process. To facilitate the analysis process, a codebook was developed, informed by the study objectives. Coding was done by two of the study team members (PT, FH) independently and involved reading through each transcript line by line and labelling chunks of text. The analysis was both deductive (driven by the objectives of the study) and inductive (allowing new themes to emerge from the data). Taking a public health approach to these issues, we drew on several public health ethics frameworks for our analysis and refer to these in the discussion section.

### Ethics approvals

A generic study protocol was tailored to the context of both countries and submitted to the Oxford Tropical Medicine Research Ethics Committee (OxTREC Ref: 552–19) and the relevant local ethics committees for ethical clearance. In Nigeria, ethics approval was obtained from the University of Ilorin Teaching Hospital (Ref: ERC/PAN/2019/07/1916) while in Burkina Faso, ethics approval was obtained from the Institutional Ethics Committee for Health Research (Ref: A35-2019/CEIRES).

## Results

The main themes that emerged in the analysis included: evidence of artemisinin and partner drug resistance, patient choice between TACTs and ACTs, the potential additional burden of TACTs and how to mitigate them, and strategies to facilitate deployment of TACTs.

Each theme is discussed below and illustrative quotes are included where relevant. These are referenced by category of stakeholder (policy maker, supplier or end-user), participant reference number and country: NG for Nigeria, and BF for Burkina Faso.

### Evidence of artemisinin and partner drug resistance

In relation to TACT deployment, mathematical modelling studies predict that TACTs could mitigate drug resistance [18]. The overall goal of the potential deployment of TACTs in Africa is to mitigate the risk of artemisinin resistance and to protect the partner drugs, thereby extending their therapeutic lifetime, which will prevent morbidity and mortality due to treatment failure. Although there was no evidence of artemisinin resistance in Burkina Faso (BF) and Nigeria (NG) at the time of collecting data for this study, the potential benefits of TACTs in preventing or delaying artemisinin resistance were discussed with the respondents during the interviews. Some respondents argued that evidence of local artemisinin resistance would be required before they would consider deploying TACTs, along with recommendation from the WHO to use TACTs. At the time of writing this paper, TACTs are not available (yet) as a commodity and therefore it is not possible for the WHO to consider and recommend TACTs as a first-line treatment. A respondent in Nigeria commented: *“Before you can bring a new drug*, *there must have been resistance and as at now I do not think there is resistance*.*” (Policy maker 10*, *NG)*.

Similar views were shared by respondents in Burkina Faso: *“I am not too much in favor of changing [to TACT] as long as ACTs are still effective*. *So we should continue to use them [ACTs] until they become resistant*. *If it does not work*, *then we can change*!*” (Supplier 21*, *BF)*.

Some respondents had opposing views. They did not think that there should be evidence of resistance locally before TACTs are introduced. A respondent at the policy level in Nigeria suggested, *“I think the population benefit of TACT is worth the effort because alternate molecules in relation to malaria have not come easily*, *every solution has taken over decades*. *Take computer for example it changes every time but it is not the same with malaria*, *it takes minimum of 10 to 20 years before you are able to get another milestone discovery and I think whatever we do to protect what we have is ethically appropriate*.*” (Policy maker 01*, *NG)*.

### Patients’ choice between TACTs and ACTs

Perspectives were divided on whether or not patients (or parents/guardians in the case of child patients) should have a choice between TACTs and ACTs while ACTs are effective. Several respondents argued that TACTs could be introduced while ACTs are still available as this will give patients and parents/guardians a choice on their treatment options rather than forcing them to take TACTs. An end-user in Nigeria said, *“They should make sure that the ones they are about to introduce are efficacious*. *I agree that they [TACTs] can be introduced because I know they may be better than before*. *But I think that the previous ones [ACTs] can still be maintained because it is not affecting us*.*” (End-user 10*, *NG)*.

Similarly, in Burkina Faso, *“We want the new to add to the old*. *We do not refuse the old…*. *like I said*, *people do not have the same blood*. *During the prescription it will be necessary to see what suits [the patient]*.*”* (*End-user 05*, *BF*).

*“You do not change a winning team! If ACTs are still effective in Burkina Faso*, *I do not know why we have to do an aggressive treatment*. *You cannot leave drugs that are good to jump to other combinations*, *I think the logic behind this abandonment is drug resistance in the present drugs*.*” (Policy maker 01, BF)*.

Others, however, supported a direct and full transition from ACTs to TACTs. Some prescribers raised concerns that a staggered transition could defeat the purpose of deploying TACTs. Some suggested that making both treatments available may not be feasible because ACTs would dwindle if TACTs prove to be more effective, and patients would no longer have access to ACTs on the market.

### Known or potential burdens and harms of deploying TACTs

Based on available data from previously conducted clinical studies on TACTs, some of the additional side effects that paediatric patients may experience compared to ACTs include an increased incidence of nausea and vomiting. These were discussed with the respondents. A respondent from Nigeria presented his views on side effects. *“The artesunate-amodiaquine has some issues of dizziness*, *drowsiness*, *nausea*, *diarrhoea*. *There are some side effects that are devastating for the patient*, *they are not fatal but some people cannot withstand it compared to some other side effects*, *I do not think it would be a hindrance*, *there are some side effects that WHO is very cautious about like if the drug has some cardiac issues or some severe liver issues*. *Normal side effects like some skin reaction*, *diarrhoea*, *vomiting… I do not think it should be a hindrance in deploying any anti-malarial whether it is 2 or 3 drugs*.*” (Policy maker 05*, *NG)*.

Perspectives from Burkina Faso were similar. A clinician/prescriber suggested that these side effects were similar to the symptoms of malaria, which are well known to community members and therefore, side effects are not per se perceived as a problem. This was reiterated by a regulator as well. *“In the villages*, *this is not a problem at all*. *There are some elderly people who believe that vomiting evacuates the germ of the disease*. *Vomiting is proof that the medicine is working in your body*.*”* (*Policy maker 04*, *BF*).

The end-users who participated in the FGDs also shared this perspective, that experiencing symptoms such as vomiting due to TACTs could be misinterpreted as the new combination drug being effective because of the effect it produces on them. *“Vomiting pushes dirt out of your belly*. *There are people when they get sick*, *they do not throw up*, *whereas it is often said in the hospital*, *that if you throw up it is good*. *There are even people who go to the hospital to pay for a product so that they can vomit and get the dirt out*. *If you have malaria*, *the dirt stays in your belly and turns yellow*. *So*, *for me*, *if the effect is vomiting*, *then it is good*.*”* (*End-user 03*, *BF)*.

Another important potential burden of deploying TACTs identified by the stakeholders were the costs of TACTs. Almost all respondents mentioned cost as a potential limiting factor to the deployment of TACTs. While some respondents suggested that TACTs should be provided to patients free of charge, others suggested that the cost of the new drug will not be a problem as long as it is effective. While the cost-benefit of TACTs cannot be determined until the clinical studies have been completed and the product is developed, most of the respondents suggested that the cost should be similar or lower to the cost of current ACTs in the market. *“If the price is slightly more than that of the current ACT*, *sir*, *it is dead on arrival*.*” (Policy maker 10*, *NG)*.

### Strategies to minimise potential burdens and harms of TACTs

In the previous section, we identified two potential burdens: additional side effects and cost.

Some study respondents (nurses, clinicians) suggested that the additional side effects that patients are likely to experience could be minimized by including an anti-emetic agent. *“If the side effects are limited to nausea and vomiting*, *I do not think that it is too much*, *there are drugs that can be taken to prevent those side effects*.*” (Supplier 01*, *NG)*. However, given that obtaining these additional drugs will likely become an out-of-pocket expense, concerns were raised regarding the cost implication of asking patients to take other medications to reduce these side effects. To these respondents, the deployment of TACTs should not create additional financial burdens on patients if the ultimate goal is the public health benefit of preventing or delaying drug resistance.

Regulators and prescribers in particular highlighted the need for proper pharmacovigilance systems to support the deployment of TACTs in Africa. *“I think that if you want to deploy the TACTs*, *I think it will be better to wake up or energize the pharmacovigilance system so that you can capture all these cases of vomiting even if it is minor*, *it affects the adherence of the medication*.*” (Policy maker 01*, *BF)*.

To address the issue of costs of the TACTs per se, respondents suggested that the cost should be either comparable or lower than the cost of current ACTs. Policy makers and suppliers suggested that key actors such as the WHO and the Global Fund to Fight Aids, Tuberculosis and Malaria should play a role in determining the costs of TACTs before their deployment. They emphasised that in order for TACTs to achieve their public health goals, they need to be affordable, acceptable and accessible to communities that need them. Drawing on lessons learnt during the past transition from monotherapies to ACTs, a regulator in Nigeria narrated steps that were taken to promote public acceptance and to address affordability and accessibility issues in the following narrative:

*“ACTs were moved from being prescription drug into being OTCs (over the counter) so that alone facilitated access…Global Fund that ran the project paid 95% to manufacturers then we got private sector people to pay 5%*. *We agreed not to sell the drug above 100 naira (USD 0*.*24)*, *if they [private sector] sell more than that they would be removed from the programme… we got waivers for private sector people to be able to bring in drugs without tax*.*” (Policy maker 01*, *NG)*.

### Strategies to facilitate introduction and deployment of TACTs

For TACTs to be accepted by the community, respondents suggested that raising community awareness and public engagement is key: *“As I had already talked about raising awareness*, *so I think that we need to make advertisements*, *press releases*, *and theatres [shows] so that people can see if the specialists themselves are the ones who are speaking*, *so that people will not refuse*.*” (End-user 04*, *BF)*.

Most participants agreed that engaging local communities early and before the deployment stage would help to address additional potential burdens.

According to a policy level respondent in Burkina Faso, *“Actually*, *ethics is all about the integrity of the human being*, *we need to know the ins and outs before we can introduce the drug*. *Socio-cultural considerations*: *you have to take these considerations into account*, *otherwise people will never accept your products if it is going to upset their habits*.*” (Policy maker 10*, *BF)*.

Various engagement strategies were highlighted by end-users as key facilitators of the deployment process. These activities could serve as a platform to maximise listening to community views in order to consider their concerns during the deployment process. Engagement with communities could also aid effective deployment by rendering the information (e.g. benefits of TACT, importance of compliance, additional side effects of TACTs) context specific and in a format accessible to communities. Some suggestions included organising community meetings, using local radios, developing information leaflets and posters.

Respondents also mentioned the importance of product quality and packaging in the deployment of TACTs. A policy maker from Burkina Faso said, *“If the presentation of the product is of quality*, *the packaging*, *the container*, *the content is well presented and if you add ‘made in Burkina Faso’*, *the product will be accepted*.*” (Policy maker 04*, *BF)*.

Other stakeholders suggested the importance of building the capacity of health workers to enable them to serve as educators to their patients. *“There is need for capacity building of health workers on TACTs*, *because if they understand [them] they can explain to the patients*.*” (Policy maker 02*, *NG)*.

While the development and deployment of TACTs were considered important for malaria control and elimination, respondents also emphasized the ongoing need to focus on other malaria preventive measures such as the use of insecticide-treated bed nets.

## Discussion

Our study sought to answer the following question: ‘*what are the ethical considerations for switching to TACTs as first-line therapy for the treatment of malaria*?*’*. The main ethical considerations included: should TACTs be deployed in the absence of evidence of artemisinin resistance locally, should patients have the option to choose between TACTs and ACTs while ACTs are still effective, and should current patients take on additional harms and burdens for the sake of future patients, and if yes, what extent of these additional burdens would be ethically justifiable.

The question of whether TACTs should be first-line therapy for the treatment of malaria in African countries can be analysed from a public health ethics lens, which is a set of moral considerations related to public health. Public health has primarily the goal of improving health of populations, which should be weighed against the impact of public health interventions on the individual [24–26]. With regard to public health interventions, according to Childress *et al*, relevant ethical considerations include effectiveness, proportionality, necessity, least infringement and public justification [25]. Applying this for a proposed public health programme the authors suggest, ‘public health agents have to consider whether any proposed programme will be likely to realize the public health goal that is sought (effectiveness), whether probable benefits will outweigh the infringed general moral considerations (proportionality), whether the policy is essential to realize the end (necessity), whether it involves the least infringement possible consistent with realizing the goal that is sought (least infringement), and whether it can be publicly justified’ [25]. According to Kass, and Upshur, the potential burdens and harms of the public health programme should be identified in advance and minimised if possible, and the programme should be implemented in a transparent manner and fairly without discrimination [24, 26].

It has been suggested that deploying TACTs in Africa has the potential to delay or prevent artemisinin resistance, thus providing a benefit at the community level, while ensuring clinical benefits for individual patients [17, 20, 22, 33]. This study sought to explore the perspectives of stakeholders within the health policy and healthcare systems of Burkina Faso and Nigeria on the key ethical considerations to be anticipated and addressed in the deployment of TACTs in Africa. Our study exemplified a mixture of ethical issues and factors relevant to policy and implementation. Our findings complement a recently reported study on regulatory and market positioning considerations of potentially deploying TACTs in Africa [28].

In terms of changing policy to use TACTs as first-line treatment in Nigeria and Burkina Faso, respondents suggested that it will depend on how imminent the threat of resistance is on the continent. Evidence to support how effective (effectiveness and necessity principles) TACTs would be in achieving the goal of preventing or mitigating the risks of drug resistance will therefore be key in deciding when and how TACTs should be deployed in Africa.

At the time of conducting data collection for this study, there were no confirmed reports yet of artemisinin resistance in the countries involved in this study; Burkina Faso and Nigeria, and currently the WHO recommends ACTs for the treatment of uncomplicated *Plasmodium falciparum* malaria [2]. Our study suggested that willingness to change national malaria policies from ACTs to TACTs would depend on evidence to support this change, steering from the WHO and funding from the Global Fund for Aids, Tuberculosis and Malaria. Ongoing clinical studies and mathematical modeling studies will aim to provide evidence to guide national malaria control programmes to make decisions on a potential change in their national malaria treatment policies [16].

Given that ACTs are still effective in most of Africa, some study respondents suggested the deployment of TACTs should be staggered over a period of time. Other respondents disagreed and were in favor of an immediate switch. Making both ACTs and TACTs available also gives patients a choice of treatment options and promotes patients’ autonomy and adheres to the public health ethics principle of “least restrictive or coercive means” or “least infringements” [24, 25]. However, as discussed previously [23], giving patients a choice between ACTs and TACTs could potentially defeat the ultimate aim of addressing the potential risks of artemisinin resistance in Africa, which could have potentially severe consequences for malaria control in Africa, and mainly affecting children. This is a familiar dilemma in public health: to what extent can public health interventions infringe on individual liberties such as imposing mandatory screening, mass drug administrations and immunizations for the sake of the health of the entire population [26, 34]? It is recommended that for any such infringements, public health agents should explain and justify the infringements to the affected parties [25].

While deployment of TACTs could benefit populations by preventing or delaying artemisinin resistance, there is no immediate *additional* benefit to individual patients in regions where ACTs are still fully effective. A key issue related to this are the additional side effects of TACTs over ACTs, which would be experienced mainly by children. With TACTs, patients (including children) could experience side effects of three rather than two antimalarial drugs, whereas there is no additional immediate benefit for the individual patient. Clinical studies conducted to date (mainly in adults from Asia) suggest that there could be a slight increase in vomiting as a result of the additional drug in the combinations [22]. This challenges the best interest principle in terms of clinical treatment of paediatric patients [23]. Clinicians, parents and society are ethically required to adhere to the best interest principle as a guide when considering the appropriateness of the specific therapy. This is in accordance with the United Nations Conventions for the Rights of the Child that emphasizes that the child’s best interest is the primary consideration in all actions concerning children [35].

The counter argument to that is the duty of easy rescue which states that if we can do something that would benefit others significantly at a small cost to us, we have a moral obligation to do it. This has been famously illustrated by Singer through his example of the drowning child, “if I walk past a pond and notice a child drowning in it, I have a moral obligation to save the child even if this means getting my clothes muddy, but this is insignificant, while death of the child would presumably be a very bad thing” [36]. However, although Singer was not explicit, it can be assumed that he was mainly referring to competent adults who can make decisions for themselves.

Notwithstanding these ethical tensions, best interest versus duty of easy rescue, there are also practical considerations related to increased side effects of TACTs over ACTs. While these additional side effects are generally considered minimal, they could deter parents from giving TACTs to their children [37]. This is consistent with previous studies documenting the experience of several malaria-endemic countries indicating that a perceived increase of side effects affected the uptake of ACTs following the change in malaria drug policies from chloroquine and sulfadoxine-pyramethamine to ACTs [38, 39]. While some respondents recommended that patients could be given other medication to reduce the side effects, others suggested that patients could perceive experiencing these side effects as an indication that TACTs work. Although this latter view could facilitate the deployment process, it would be important to address community perceptions about TACTs and side effects in general that may be inaccurate. It is also important to address their views on vomiting as a side effect in particular, and educate them on how and when to re-dose. Of particular importance in the drug development process is manufacturing paediatric tablets that are dispersible, taste-masking and fixed-dose combination of drugs in order to reduce the number of tablets.

Another issue emerging from our data is affordability of TACTs compared with current ACTs. Most respondents suggested that community acceptance of TACTs would depend on the cost of TACTs and recommended that TACTs should not be more expensive than ACTs, to patients. Several studies have reported affordability as a key limiting factor when considering access to effective drugs, to patients who need them most [40, 41]. This suggests that if TACTs are more costly than current ACTs, most patients are likely to use other antimalarial drugs thus defeating the aim of delaying or reducing artemisinin resistance. As outlined by public health ethics frameworks, the burdens of TACTs such as additional cost to end-users should be minimized [26]. National malaria control programmes would need to advocate for TACTs to be covered under national health insurance schemes, provided for free under donor programmes, or negotiate with drug developers to make these drugs more affordable to patients [28].

Respondents emphasized the need for extensive community, public and stakeholder engagement regarding TACTs before deployment in African communities. This is in line with the transparency principle identified by Upshur requiring that all legitimate stakeholders are involved in the decision-making process and have input in the implementation of TACTs [24]. Engagement is also important to avoid some of the challenges that many African countries encountered during the change from monotherapies to ACTs in the early 2000s [42]. Strategies and engagement channels such as the use of local radio, conducting art and theatre events and collaborating with community leaders to disseminate information about TACTs to community members could support the engagement process. These have been successfully trialed for malaria programmes in several countries [43–48]. The potential of communities perceiving TACTs as a new drug was often mentioned in study responses suggesting that there is a need to use innovative methods to explain that TACTs are not new drugs but a combination of existing drugs that have been used for many years, and that the immediate efficacy is the same (in the absence of ACT failure). Information about TACTs should also mention that the addition of a third drug protects the drugs, which means they can be used for a longer time, providing a longer lasting option for future patients.

Respondents furthermore emphasized the need to focus on other malaria preventive measures such as the use of insecticide-treated bed nets. These views suggest that there will be a need for more investments to align the deployment of TACTs with other malaria control strategies and ensure that deployment of TACTs does not deviate the attention or funding allocated to these important on-going malaria control activities.

### Strengths and limitations

The main strength of the study is that we preemptively and proactively sought stakeholder views to inform the potential deployment of TACTs to avoid potential pitfalls during their introduction and deployment. However, the study was conducted in only two countries in west Africa; Burkina Faso and Nigeria, and thus the perspectives reported in this paper would not represent the whole of the African sub-region. We also acknowledge that our study may not be exhaustive in finding all relevant factors that influence policy making. Additionally, given that TACTs are not available on the market yet, and the cost is still unknown, most of the discussions were hypothetical and perspectives are likely to change when a product is ready for deployment. Similarly, the efficacy of ACTs could also be different than at present when the TACTs are ready for deployment. This is demonstrated in a recent report from Burkina Faso suggesting treatment failure following artemether-lumefantrine and additional reports of artemisinin partial resistance associated with delayed parasite clearance in Rwanda and Uganda [7, 11, 12].

Despite these limitations, the study highlights key ethical issues that can help inform strategies for the deployment of TACTs in African countries. We encourage similar studies in other African countries to build relevant evidence to support policy makers and implementers.

## Conclusions

This study sought to explore the perspectives of stakeholders within the healthcare systems of Burkina Faso and Nigeria on the key ethical considerations to be anticipated and addressed in the potential deployment of TACTs in Africa. The main ethical considerations, for which responses were not unanimous, are whether or not to deploy ACTs in the absence of artemisinin resistance locally, whether or not patients should be able to choose between TACTs and ACTs while ACTs are still effective, and whether and how much current patients should take on additional burdens for the sake of future patients.

## References

[1] Brentlinger PE. Health, human rights, and malaria control: historical background and current challenges. Health Hum Rights. 2006;9(2):10–38. 17265753

[2] World Health Organisation 2022. WHO Guidelines for Malaria 2022. Accessed 20th May 2022.

[3] Bosman A, Mendis KN. A major transition in malaria treatment: the adoption and deployment of artemisinin-based combination therapies. Am J Trop Med Hyg. 2007;77(6 Suppl):193–7. 18165492

[4] Malik EM, Mohamed TA, Elmardi KA, Mowien RM, Elhassan AH, Elamin SB, et al. From chloroquine to artemisinin-based combination therapy: the Sudanese experience. Malar J. 2006;5:65. doi: 10.1186/1475-2875-5-65 16879742 PMC1590042

[5] Nanyunja M, Nabyonga Orem J, Kato F, Kaggwa M, Katureebe C, Saweka J. Malaria treatment policy change and implementation: the case of Uganda. Malar Res Treat. 2011;2011:683167. doi: 10.4061/2011/683167 22312571 PMC3265287

[6] Asua V, Vinden J, Conrad M, Legac J, Kigozi S, Kamya M, et al. Changing Molecular Markers of Antimalarial Drug Sensitivity across Uganda. Antimicrobial Agents and Chemotherapy. 2018;63. 10.1128/AAC.01818-18 PMC6395896 30559133

[7] Uwimana A, Legrand E, Stokes BH, Ndikumana JM, Warsame M, Umulisa N, et al. Emergence and clonal expansion of in vitro artemisinin-resistant Plasmodium falciparum kelch13 R561H mutant parasites in Rwanda. Nat Med. 2020;26(10):1602–8. doi: 10.1038/s41591-020-1005-2 32747827 PMC7541349

[8] Ikeda M, Kaneko M, Tachibana SI, Balikagala B, Sakurai-Yatsushiro M, Yatsushiro S, et al. Artemisinin-Resistant Plasmodium falciparum with High Survival Rates, Uganda, 2014–2016. Emerg Infect Dis. 2018;24(4):718–26. doi: 10.3201/eid2404.170141 29553316 PMC5875287

[9] Kayiba NK, Yobi DM, Tshibangu-Kabamba E, Tuan VP, Yamaoka Y, Devleesschauwer B, et al. Spatial and molecular mapping of Pfkelch13 gene polymorphism in Africa in the era of emerging Plasmodium falciparum resistance to artemisinin: a systematic review. Lancet Infect Dis. 2021;21(4):e82–e92. doi: 10.1016/S1473-3099(20)30493-X 33125913

[10] Uwimana A, Umulisa N, Venkatesan M, Svigel SS, Zhou Z, Munyaneza T, et al. Association of Plasmodium falciparum kelch13 R561H genotypes with delayed parasite clearance in Rwanda: an open-label, single-arm, multicentre, therapeutic efficacy study. Lancet Infect Dis. 2021;21(8):1120–8. doi: 10.1016/S1473-3099(21)00142-0 33864801 PMC10202849

[11] Balikagala B, Fukuda N, Ikeda M, Katuro OT, Tachibana SI, Yamauchi M, et al. Evidence of Artemisinin-Resistant Malaria in Africa. N Engl J Med. 2021;385(13):1163–71. doi: 10.1056/NEJMoa2101746 34551228

[12] Gansane A, Moriarty LF, Menard D, Yerbanga I, Ouedraogo E, Sondo P, et al. Anti-malarial efficacy and resistance monitoring of artemether-lumefantrine and dihydroartemisinin-piperaquine shows inadequate efficacy in children in Burkina Faso, 2017–2018. Malar J. 2021;20(1):48. doi: 10.1186/s12936-021-03585-6 33468147 PMC7816451

[13] Dimbu PR, Horth R, Cândido ALM, Ferreira CM, Caquece F, Garcia LEA, et al. Continued Low Efficacy of Artemether-Lumefantrine in Angola in 2019. Antimicrobial Agents and Chemotherapy. 2021;65(2):e01949–20. doi: 10.1128/AAC.01949-20 33168604 PMC7849008

[14] Rasmussen C, Ringwald P. Is there evidence of anti-malarial multidrug resistance in Burkina Faso? Malar J. 2021;20(1):320. doi: 10.1186/s12936-021-03845-5 34281562 PMC8287766

[15] Rasmussen C, Ringwald P. Continued Low Efficacy of Artemether-Lumefantrine in Angola? Antimicrob Agents Chemother. 2021;65(6). 10.1128/AAC.00220-21 PMC8316127 33753339

[16] van der Pluijm RW, Amaratunga C, Dhorda M, Dondorp AM. Triple Artemisinin-Based Combination Therapies for Malaria—A New Paradigm? Trends Parasitol. 2021;37(1):15–24. doi: 10.1016/j.pt.2020.09.011 33060063

[17] White NJ. Triple artemisinin-containing combination anti-malarial treatments should be implemented now to delay the emergence of resistance. Malar J. 2019;18(1):338. doi: 10.1186/s12936-019-2955-z 31581941 PMC6777025

[18] Kunkel A, White M, Piola P. Novel anti-malarial drug strategies to prevent artemisinin partner drug resistance: A model-based analysis. PLoS Comput Biol. 2021;17(3):e1008850. doi: 10.1371/journal.pcbi.1008850 33764971 PMC8023453

[19] Hanboonkunupakarn B, White NJ. Advances and roadblocks in the treatment of malaria. Br J Clin Pharmacol. 2020. doi: 10.1111/bcp.14474 32656850 PMC9437935

[20] Veiga MI, Dhingra SK, Henrich PP, Straimer J, Gnadig N, Uhlemann AC, et al. Globally prevalent PfMDR1 mutations modulate Plasmodium falciparum susceptibility to artemisinin-based combination therapies. Nat Commun. 2016;7:11553. doi: 10.1038/ncomms11553 27189525 PMC4873939

[21] Cheah PY, Day N, Parker M. 2020. Ethics and antimalarial drug resistance. In Ethics and drug resistance: Collective responsibility for global public health. Cham:Springer.

[22] van der Pluijm RW, Tripura R, Hoglund RM, Pyae Phyo A, Lek D, Ul Islam A, et al. Triple artemisinin-based combination therapies versus artemisinin-based combination therapies for uncomplicated Plasmodium falciparum malaria: a multicentre, open-label, randomised clinical trial. Lancet. 2020;395(10233):1345–60. doi: 10.1016/S0140-6736(20)30552-3 32171078 PMC8204272

[23] Tindana P, de Haan F, Amaratunga C, Dhorda M, van der Pluijm RW, Dondorp AM, et al. Deploying triple artemisinin-based combination therapy (TACT) for malaria treatment in Africa: ethical and practical considerations. Malar J. 2021;20(1):119. doi: 10.1186/s12936-021-03649-7 33639946 PMC7910789

[24] Upshur RE. Principles for the justification of public health intervention. Can J Public Health. 2002;93(2):101–3. doi: 10.1007/BF03404547 11968179 PMC6979585

[25] Childress JF, Faden RR, Gaare RD, Gostin LO, Kahn J, Bonnie RJ, et al. Public health ethics: mapping the terrain. J Law Med Ethics. 2002;30(2):170–8. doi: 10.1111/j.1748-720x.2002.tb00384.x 12066595

[26] Kass NE. An ethics framework for public health. Am J Public Health. 2001;91(11):1776–82. doi: 10.2105/ajph.91.11.1776 11684600 PMC1446875

[27] Tindana P, de Haan F, Mokuolu OA, Guissou R, Bolarinwa OA, Ouedraogo JB, et al. Ethical, regulatory and market related aspects of deploying triple artemisinin-based combination therapies for malaria treatment in Africa: A study protocol. Wellcome Open Res. 2021;6:75. doi: 10.12688/wellcomeopenres.16065.1 34458588 PMC8378406

[28] de Haan F, Bolarinwa OA, Guissou R, Tou F, Tindana P, Boon WPC, et al. To what extent are the antimalarial markets in African countries ready for a transition to triple artemisinin-based combination therapies? PLoS One. 2021;16(8):e0256567. doi: 10.1371/journal.pone.0256567 34464398 PMC8407563

[29] Gansane A, Nebie I, Soulama I, Tiono A, Diarra A, Konate AT, et al. [Change of antimalarial first-line treatment in Burkina Faso in 2005]. Bull Soc Pathol Exot. 2009;102(1):31–5. 19343918 10.3185/pathexo3235

[30] Federal Republic of Nigeria: National Antimalarial Treatment Policy, FMOH, National malaria and Vector Control Division, Abuja, Nigeria, 2005.

[31] Federal Ministry of Health, National Malaria Control Programme, Abuja, Nigeria. Strategic Plan 2009–2013, 2008, Federal Ministry of Health, National Malaria Control Programme.

[32] de Haan F, Bolarinwa OA, Guissou R, et al. 2021: Interview guides in English and French. PLOS ONE. Dataset. 10.1371/journal.pone.0256567.s001.

[33] Plowe CV. The evolution of drug-resistant malaria. Trans R Soc Trop Med Hyg. 2009;103 Suppl 1:S11–4. doi: 10.1016/j.trstmh.2008.11.002 19084883 PMC2723787

[34] Cheah PY, White NJ. Antimalarial mass drug administration: ethical considerations. Int Health. 2016;8(4):235–8. doi: 10.1093/inthealth/ihw027 27481834 PMC4967848

[35] United Nations Children’s Fund 1989. The convention on the rights of the child. https://www.unicef.org/child-rights-convention/convention-text. Accessed 4 August 2022.

[36] Singer P. Famine, affluence, and morality. Philosophy and Public Affairs 1972; 1 (3): 229–243.

[37] de Haan F, Boon WPC, Amaratunga C, Dondorp AM. Expert perspectives on the introduction of Triple Artemisinin-based Combination Therapies (TACTs) in Southeast Asia: a Delphi study. BMC Public Health. 2022;22(1):864. doi: 10.1186/s12889-022-13212-x 35490212 PMC9055751

[38] Amin AA, Zurovac D, Kangwana BB, Greenfield J, Otieno DN, Akhwale WS, et al. The challenges of changing national malaria drug policy to artemisinin-based combinations in Kenya. Malar J. 2007;6:72. doi: 10.1186/1475-2875-6-72 17535417 PMC1892027

[39] Maslove DM, Mnyusiwalla A, Mills EJ, McGowan J, Attaran A, Wilson K. Barriers to the effective treatment and prevention of malaria in Africa: A systematic review of qualitative studies. BMC Int Health Hum Rights. 2009;9:26. doi: 10.1186/1472-698X-9-26 19852857 PMC2782321

[40] Mutabingwa TK. Artemisinin-based combination therapies (ACTs): best hope for malaria treatment but inaccessible to the needy! Acta Trop. 2005;95(3):305–15. doi: 10.1016/j.actatropica.2005.06.009 16098946

[41] Chuma J, Okungu V, Molyneux C. Barriers to prompt and effective malaria treatment among the poorest population in Kenya. Malar J. 2010;9:144. doi: 10.1186/1475-2875-9-144 20507555 PMC2892503

[42] Mutero CM, Kramer RA, Paul C, Lesser A, Miranda ML, Mboera LE, et al. Factors influencing malaria control policy-making in Kenya, Uganda and Tanzania. Malar J. 2014;13:305. doi: 10.1186/1475-2875-13-305 25107509 PMC4249611

[43] Lim R, Tripura R, T JP, Sareth M, Sanann N, Davoeung C, et al. Drama as a community engagement strategy for malaria in rural Cambodia. Wellcome Open Res. 2017;2:95. doi: 10.12688/wellcomeopenres.12594.2 29062919 PMC5645711

[44] Lim R, Peto TJ, Tripura R, Cheah PY. Village Drama Against Malaria. Lancet. 2016;388(10063):2990. doi: 10.1016/S0140-6736(16)32519-3 27998527

[45] Sanga G, Jao I, Mumba N, Mwalukore S, Kamuya D, Davies A. Always leave the audience wanting more: An entertaining approach to stimulate engagement with health research among publics in coastal Kenya through ’Magnet Theatre’. Wellcome Open Res. 2021;6:2. doi: 10.12688/wellcomeopenres.16461.2 33824910 PMC8008348

[46] Adhikari B, Pell C, Cheah PY. Community engagement and ethical global health research. Glob Bioeth. 2020;31(1):1–12. doi: 10.1080/11287462.2019.1703504 32002019 PMC6968663

[47] Nguon C, Dysoley L, Davoeung C, Sovann Y, Sanann N, Sareth M, et al. Art and theatre for health in rural Cambodia. Glob Bioeth. 2018;29(1):16–21. doi: 10.1080/11287462.2017.1411762 29249920 PMC5727450

[48] Callery JJ, Sanann N, Tripura R, Buntau T, Peto TJ, Kunthea P, et al. Engaging ethnic minority communities through performance and arts: health education in Cambodian forest villages. Int Health. 2021;13(2):188–95. doi: 10.1093/inthealth/ihaa076 33038893 PMC7902271

